# Chromium Speciation in Wastewater and Sewage by Solid-Phase Extraction Using a New Diphenylcarbazone-Incorporated Resin

**DOI:** 10.1007/s11270-016-2974-0

**Published:** 2016-07-29

**Authors:** Barbara Leśniewska, Anna Jeglikowska, Beata Godlewska-Żyłkiewicz

**Affiliations:** Institute of Chemistry, University of Bialystok, K. Ciołkowskiego 1K, 15-245 Bialystok, Poland

**Keywords:** Chromium(III), Separation, Functionalized resin, FAAS, 1,5-Diphenylcarbazone, Environmental analysis

## Abstract

A new procedure for the determination of chromium species in polluted environmental samples by flame atomic absorption spectrometry was developed in this work. A new material containing 1,5-diphenylcarbazone included in a polymeric matrix was prepared and employed as a solid-phase extraction material for selective separation of Cr(III) ions under dynamic conditions. Chromium(III) ions were retained on this sorbent with high efficiency and repeatability (95 %, RSD = 1 %) from solutions with pH 9.0. The quantitative recovery of analyte was obtained with 0.1 mol L^−1^ EDTA. The concentration of Cr(VI) ions was calculated from the difference between the concentration of total chromium and Cr(III) ions. The prepared sorbent exhibits good chemical and mechanical stability, sorption capacity and selectivity towards Cr(III) ions in the presence of Cu(II), Ni(II), Mn(II) and Ca(II) ions. The accuracy of the separation method was proved by analysis of reference material of wastewater RES 10.2. The developed procedure was applied for chromium speciation analysis in municipal sewage samples.

## Introduction

Chromium is considered a priority pollutant by the US Environmental Protection Agency due to significant quantities of its production and frequent occurrence in water systems (EPA 2014). Total chromium emission in 2013 in the European Union (EU-28) was 336 Mg, while in Poland was 47 Mg (EEA Technical report 2015). The wide use of chromium in steel and electroplating industry, leather tanning, production of alloys and pigments leads to the discharge of significant quantities of liquid or solid wastes containing chromium into the environment, which pollutes groundwater and surface water, soils and plants (Kotaś and Stasicka 2000; Metze et al. 2005; Hoet 2005). The concentrations of chromium in wastewaters (such as electroplating or leather tanning wastewaters) are usually in the range of 3–30 mg L^−1^ for Cr(VI) and 5–100 mg L^−1^ for total chromium. In Poland, according to the Ordinance of the Minister of Environment (2014) concerning the conditions for spreading sewage into the water or soil, the maximal concentration of chromium in sewage cannot exceed 0.05–0.5 mg L^−1^ of Cr(VI) and 0.5–1 mg L^−1^ of total chromium, depending on the type of industry.

Two most stable oxidation states of chromium, Cr(III) and Cr(VI), exhibit different biological activity and toxic effect on living organisms. Cr(III) is an essential element for the proper functioning of living organisms, while Cr(VI) has an adverse effect on living organisms. Owing to its high toxicity, this metal constitutes a serious risk for health, whereas chronic exposure to it, even at low concentrations, may produce mutagenesis and carcinogenesis (IARC 1990; Report on Carcinogens 2014). Compounds of Cr(III) and Cr(VI) differ also in mobility under environmental conditions. More mobile species of Cr(VI) can undergo redox transformation forming labile and more stable species of Cr(III), Hence, speciation analysis of chromium in various environmental compartments and in humans is essential.

In speciation analysis of chromium by the spectrophotometric method, complexes of chromium at different oxidation states have been exploited. Spectrophotometric detection requires the transformation of one of the chromium species, most often Cr(VI), into a colourful form, which is able to absorb visible (vis) radiation. The most popular methods are based on the formation of complexes of Cr(VI) with 1,5-diphenylcarbazide (DPC) (Mulaudzi et al. 2002; Ma et al. 2014), sodium diphenylamine sulfonate (Yuan et al. 2008) and chromotropic acid (Themelis et al. 2006) showing absorption maxima at *λ*_max_ = 540, 550 and 370 nm, respectively. Limits of detection of the proposed methods were 23 μg L^−1^ (Mulaudzi et al. 2002; Ma et al. 2014), 22 μg L^−1^ (Yuan et al. 2008) and 1 μg L^−1^ (Themelis et al. 2006).

In the reaction of Cr(VI) with DPC, the ligand is oxidized by chromate ions to 1,5-diphenylcarbazone (DPCO) leading to the formation of a soluble red-violet complex of Cr(III)-DPCO. Although literature review shows that this method is most often used for the selective determination of Cr(VI), it suffers from the presence of interfering compounds, particularly Cu(II), Mo(VI), Fe(III), V(V) and Hg(II), which can react with the complexing agent giving positive interferences. A large excess of DPC is essential, as compounds present in the sample may consume the reagent. On the other hand, humic compounds present in the sample, including species that absorb at 540 nm (e.g., released from soil), interfere with the correct determination of Cr(VI) (Pettine and Capri 2005). Moreover, the Cr(III)-DPCO complex is photo-labile leading to a fast decrease of analytical signal. It was observed during measurement of Cr(III)-DPCO in solution by a thermal lens spectrometry (TLS) (Madzgalj et al. 2008). Due to these reasons, direct application of this method to real sample analysis is frequently restricted because of the effect of sample matrix compositions and relatively low sensitivity.

In order to obtain reliable results of chromium speciation analysis, enrichment and separation techniques, such as coprecipitation (Soylak and Kizil 2013), liquid-liquid extraction (Pena et al. 2008; Unsal et al. 2014), solid-phase extraction (Leśniewska et al. 2012; Leśniewska et al. 2015; Tunceli and Turker 2002; Narin et al. 2002) or chromatography (Stanisławska et al. 2013; Barałkiewicz et al. 2013), coupled to species non-specific detection techniques, such as flame atomic absorption spectrometry (FAAS) (Table 1) or electrothermal atomic absorption spectrometry (ETAAS) or inductively coupled plasma mass spectrometry (ICP-MS), have been proposed (Table 1). These methods have been extensively discussed in several review papers (Zhao et al. 2012; Pyrzyńska 2012; Namieśnik and Rabajczyk 2012; Ščančar and Milačič 2014). The solid-phase extraction (SPE) separation methods used for chromium speciation are mostly based on the separation and determination of one of the chromium forms (Cr(III) or Cr(VI)). The content of the second form is determined after its oxidation/reduction or as a difference between total chromium and the initially determined form. Commercial or modified/functionalized materials such as chelating resins, anion or cation exchangers and adsorptive resins are typically used as solid sorbents.

**Table 1:** Comparison of some methods for determination of chromium species by flame atomic absorption spectrometry in natural water and wastewater

Separated form of analyte	Separation method	Sorption capacity (mg g^−1^)	PF or PF^a^	LOD or LOD^b^ (μg L^−1^)	RSD, %	Analyzed samples	Ref.
Cr(III)	Coprecipitation on Nd(OH)_3_		100	2.1		Natural water	Soylak and Kizil (2013)
Cr(III)-Sudan blue	DLLE		80^a^	1.7^b^	6.2	Natural water, wastewater, food, hair	Unsal et al. (2014)
Cr(III)-8HQ	SPE: graphene	24.8	5^a^	12.5^b^	4.3	Tap and river water	Chang et al. (2012)
Cr(III)-dithizonate	SPE: Chromosorb 108	4.5	1.4^a^	37.3^b^	1.4–5.8	River and sea water	Tuzen and Soylak (2006)
Cr(III)	SPE: XAD 2-POx	1.1	0.25^a^	58	2.5–3.0	Electroplating wastewater, seawater	Filik et al. (2003)
Cr(III) and Cr(VI)	SPE: NDSA resin	20.861.4			0.3–1.0	Industrial wastewater	Mondal et al. (2002)
Cr(III)	SPE: *Bacillus sphaericus* loaded Diaion SP-850	7.0	1^a^	25^b^	0.3–2.9	Industrial wastewater	Tuzen et al. (2007)
Cr(III)	SPE: IIP: Cr(III)-8-HQ-St-DVB	8.5	33^a^	2.1^b^	0.5–3.4	Tap water, wastewater	Leśniewska, et al. (2015)
Cr(III)	SPE: polymer with DPCO	5.4		30	3.2–3.7	Municipal sewage, wastewater RES 10.2	This work

*DLLE* dispersive liquid-liquid microextraction, *8HQ* 8-hydroxyquinoline, *XAD 2-POx* 5-palmitoyl-8-hydroxyquinoline functionalized XAD 2, *NDSA* polystyrene divinylbenzene copolymer functionalized with 2-naphthol-3,6-disulfonic acid, *IIP: Cr(III)-8-HQ-St-DVB* Cr(III)-8-hydroxyquinoline-imprinted poly(styrene-co-divinylbenzene)

PF^a^—preconcentration factor recalculated for 10 mL of sample

LOD^b^—limit of detection calculated for ^a^

The solid-phase extraction methods using the DPC agent for chromium speciation analysis are mostly based on the formation of the Cr(III)-diphenylcarbazone complex and its retention on adsorption resin such as Amberlite XAD-16 (Tunceli and Turker 2002), Amberlite XAD-4 (Rajesh et al. 2008), Amberlite XAD-1180 (Narin et al. 2008) or Ambersorb 563 (Narin et al. 2006). The content of Cr(VI) was determined after elution of the retained complex from the column by UV-vis spectrophotometry (Rajesh et al. 2008; Narin et al. 2006) or FAAS (Tunceli and Turker 2002; Narin et al. 2002; Narin et al. 2008). Total chromium was determined after conversion of Cr(III) to Cr(VI) by oxidation with KMnO_4_ (Tunceli and Turker 2002) or K_2_S_2_O_8_ in an acidic medium (Narin et al. 2006; Narin et al. 2008). The content of Cr(III) was calculated as the difference between the total Cr content and the Cr(VI) content.

The other methodology used was physical immobilization of DPC onto C18-poly(styrene-divinylbenzene) bead surfaces. The Cr(VI), present in natural water samples, reacted with the immobilized DPC in an acidic environment (5 mol L^−1^ HNO_3_) directly on the mini-column. The retained Cr(VI) ions were then eluted by a solution of methanol in water and determined by ETAAS. The used beads were discarded from the column by delivering to waste (Long et al. 2005).

Another approach could be an application of sorbents functionalized with DPC or DPCO ligands. Multi-walled carbon nanotubes modified with DPC were used for separation of Cd(II) (Behbahani et al. 2013a), magnetic nanoparticles functionalized with DPC were used for separation of Hg(II) (Zhai et al. 2010), SBA-15-modified nanoporous silica was used for separation of Cd(II) and Cu(II) (Bagheri et al. 2012), while magnetic mesoporous silica nanoparticles coated with an ion-imprinted polymer containing DPC ligand were used for separation of Pb(II) (Aboufazeli et al. 2013). Sorbents modified with DPCO ligand, such as silica gel and ion-imprinted polymer, were used for separation and preconcentration of Hg(II) (Moghimi and Poursharifi 2011) and Pb(II) (Behbahani et al. 2013b), respectively. So far, such sorbents have not been used for chromium speciation analysis.

The aim of this work was to develop a new method for chromium speciation analysis in contaminated environmental samples, wastewater and municipal sewage, by FAAS. A new polymeric sorbent for selective separation of chromium(III) was designed and prepared. The reagent often used for spectrophotometric determination of Cr(VI) species, namely DPC, was used for the formation of a Cr(III)-1,5-diphenylcarbazone (Cr(III)-DPCO) complex; next, the complex was incorporated by non-covalent bonds into a methacrylate polymeric structure. The sorption property of obtained sorbent was evaluated under dynamic conditions after removing Cr(III) ions with EDTA solution. Finally, the polymer was used as a selective sorbent for Cr(III) ions in SPE procedure for chromium speciation analysis.

## Experimental

### Instrumentation

For determination of chromium atomic absorption spectrometry with atomization in air-acetylene flame and deuterium, background correction (Solaar M6, Thermo Electron Corporation, UK) was used. The measurements of chromium were performed at wavelength *λ* = 357.9 nm with a slit of 0.5 nm by using a chromium hollow cathode lamp (Photron, Australia) operated at 6-mA current.

The FT-IR absorption spectrum of polymer (4000–500 cm^−1^) in KBr was recorded using Thermo Nicolet Magna IR 550 Series II (Nicolet, Japan). Surface area of the polymer was measured by Brunauer, Emmett and Teller (BET) method using surface area and porosity analyzer Gemini VII 2390 (Micrometrics, USA). A scanning electron microscope SEM Inspect S50 (Hitachi, USA) was used for studies of morphology of polymer particles.

A SPE system consisting of laboratory-made glass adsorption column (internal diameter (i.d.) 9 mm) filled with 0.1 g of the polymer, PTFE tubing of i.d. 0.8 mm and a peristaltic pump Minipuls 3 (Gilson, France) was used.

The pH values of solutions were controlled with an inoLab pH Level 1 pH meter (WTW, Germany) equipped with an electrode SenTix 21 (WTW, Germany).

### Reagents and Materials

Deionized Milli-Q water (Millipore, USA) was used for the preparation of all solutions. Working standard solutions of chromium were prepared daily by appropriate dilution of the stock standard: Cr(III) (20 g L^−1^, Merck, Germany) or Cr (VI) (1.001 g L^−1^, Sigma-Aldrich, Germany). Ammonia solution (25 %) supplied by POCh (Poland) and hydrochloric acid (37 %, TraceSelect, Fluka, Germany) were used for pH adjustment. EDTA obtained from Merck (Germany) was used as a desorption agent. Nitrates(V) of copper(II), nickel(II), manganese(II) and calcium (Fluka, Germany) were used for studies of competitive ions on chromium separation. 1,5-Diphenylcarbazide (DPC, Puriss, Fluka, Germany), methacrylic acid (MAA, Sigma-Aldrich, Germany), glycol ethylene dimethacrylate (EGDMA, 98 %, Sigma-Aldrich, Germany), lauroyl peroxide (Sigma-Aldrich, Germany) and ethanol (96 %, p.a.; POCh, Poland) were used for synthesis of the polymer. De-aeration of polymerization solutions was performed under a high-purity argon atmosphere.

Reference material of wastewater from an urban treatment plant, RES 10.2 (ielab Calidad, Spain), was used for the evaluation of accuracy of the developed procedure. Sewage samples were delivered from a municipal sewage treatment plant (Bialystok, Poland).

### Preparation of Polymer and Its Characteristic

The complex of Cr(III)-1,5-diphenylcarbazone (Cr(III)-DPCO) was prepared according to the procedure described in Tunceli and Turker (2002). The prepared complex (containing 6 mg, 0.12 mmol of Cr(III)) was dissolved in 11 mL of ethanol; next, 0.85 mL (10 mmol) of MAA (functional monomer), 7.5 mL (40 mmol) of EGDMA (cross-linker) and 0.151 g lauroyl peroxide (initiator) were added. The polymerization mixture was transferred into glass polymerization ampoules, stirred for 15 min, purged with argon for 10 min, sealed and heated at 60 °C for ∼18 h. The formed polymeric block was crushed in mortar. To remove excess unreacted monomers, polymeric particles were washed with 100 mL of methylene chloride and dried at 50 °C. The Cr(III) ions were removed from portions of the polymer with 0.2 mol L^−1^ EDTA. The FT-IR (in KBr) spectrum of the polymer was registered, and the following characteristic bonds were observed: υ(–C═O) at 1721 cm^−1^, υ(–C–O) at 1252 cm^−1^, υ(–C–N) at 1144 cm^−1^, υ(–C═N) at 1637 cm^−1^ and υ(–C–H) bands at 752, 1452, 2955 and 2985 cm^−1^.

Nitrogen sorption analysis was carried out on approximately 0.3-g portions of polymers degassed for 24 h at 80 °C. The surface area of the polymer was derived from adsorption isotherms, using a BET method. The BET surface area for the polymer was 185 m^2^ g^−1^, pore volume was 0.056 cm^3^ g^−1^, and pore diameter was 1.19 nm.

**Fig. 1:**
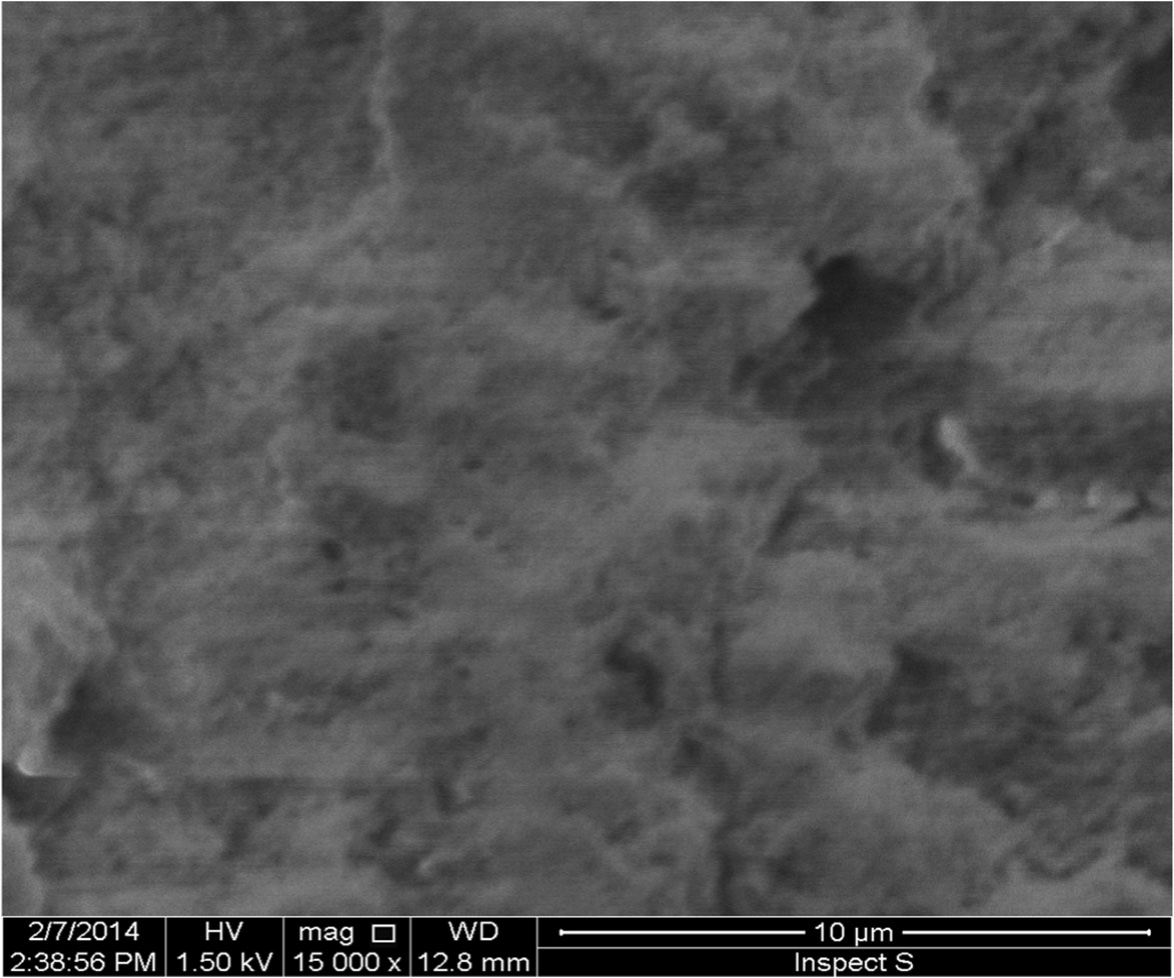
SEM image of surface particles of the polymeric resin functionalized with the DPCO ligand (×15,000 magnification)

Scanning electron microscopy (SEM) images showed that the particles display an irregular shape and small size (10–30 μm). The surface of particles of the polymer is very rough and porous (Fig. 1).

### Separation Procedure

The polymeric resin was conditioned by passing 2 mL of Milli-Q water at a flow rate of 1 mL min^−1^. The Cr(III) standards and samples were adjusted to pH 9 with ammonia and passed through the column at a flow rate of 0.7 mL min^−1^ for retention of the analyte. Two millilitres of 0.1 mol L^−1^ EDTA at a flow rate of 0.37 mL min^−1^ was used for the elution of Cr(III) ions. In order to remove Cr(VI) ions and retain matrix components of real samples, the column was rinsed with 2 mL of water before the elution step. All results are based on at least three parallel replications.

## Results and Discussion

### Optimization of the Cr(III) Separation Procedure

The suitability of the polymeric sorbent for the separation of Cr(III) ions was evaluated. The following parameters: sample pH, flow rate of sample solution through the column, a kind of eluent, its flow rate and volume were optimized.

**Fig. 2:**
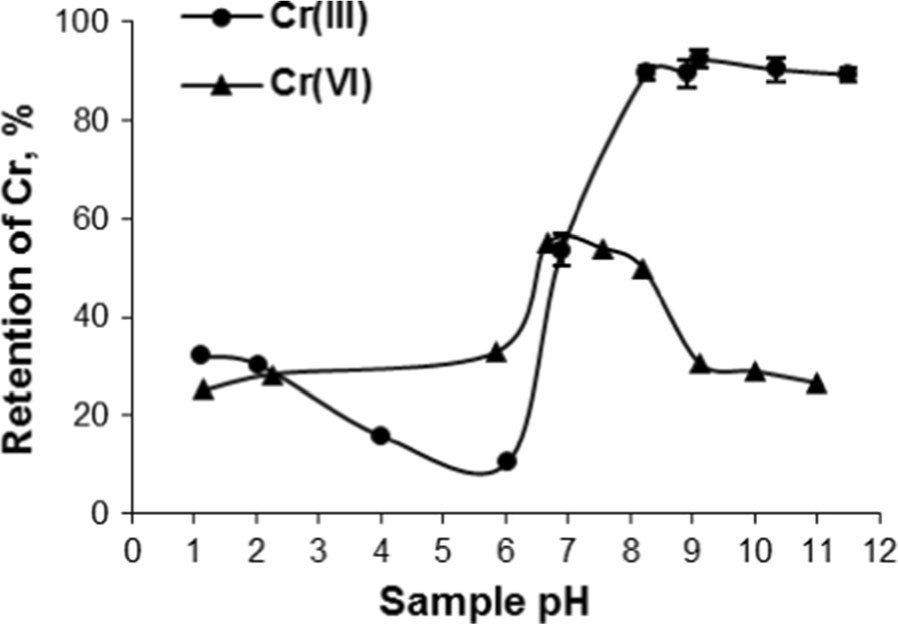
Effect of sample pH on Cr(III) and Cr(VI) retention on the polymeric resin functionalized with DPCO ligand (sample 10 μg of Cr, pH 9, flow rate 0.7 mL min^−1^)

The efficiency of retention of both chromium forms on the polymer was studied in the pH range from 1 to 12. The retention of Cr(III) (10 μg) was in the range 8–30 % in acidic solutions (pH ≤ 6), rapidly increased to 50 % in solutions of pH 7 and exceeded 90 % in solutions of pH ≥8 (Fig. 2). The retention of Cr(VI) (10 μg) from samples of pH 1–8 was in the range 30–50 % and decreased to 20 % at pH >9. This trend is probably due to the effect of unspecific bonding of this form to the functionalized sorbent. The flow rate of Cr(III) solutions in the range of 0.45 to 1.0 mL min^−1^ practically did not affect the efficiency of sorption (92–91 %) and decreased slowly to 83 % at 1.2 mL min^−1^. Hence, a flow rate of 0.7 mL min^−1^ was chosen for subsequent experiments.

**Fig. 3:**
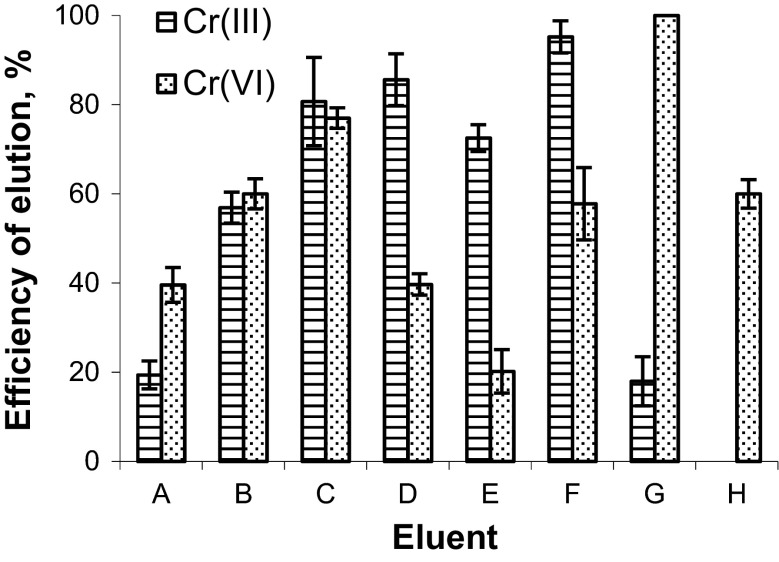
Efficiency of the elution of Cr(III) and Cr(VI) from the polymeric resin functionalized with DPCO ligand with different stripping agents: *A* 0.5 mol L^−1^ HNO_3_; *B* 2.0 mol L^−1^ HNO_3_; *C* 2.0 mol L^−1^ CH_3_COOH; *D* 0.3 mol L^−1^ HCl; *E* 0.3 mol L^−1^ thiourea in 0.3 mol L^−1^ HCl; *F* 0.2 mol L^−1^ EDTA; *G* 2.0 mol L^−1^ NH_3_aq; *H* Milli-Q water (volume 2 mL, flow rate 0.37 mL min^−1^)

Various stripping agents, mineral and organic acids and complexing agents, were tested for elution of Cr(III) and Cr(VI) ions from the sorbent (Fig. 3). The studies showed that the most effective elution of Cr(III) was obtained with 0.2 mol L^−1^ EDTA, while its elution with H_2_O was negligible. The elution of Cr(VI) with these two agents was at the level of 60 %. In order to improve the selectivity of the procedure, the column was rinsed with water prior to the elution of Cr(III) ions. This wash-up step removed most of the Cr(VI) ions from the column.

The effect of the concentration of EDTA and its flow rate on the recovery of Cr(III) (*n* = 3) was also studied. The efficiency of Cr(III) elution raised from 75 ± 6 to 101 ± 1 % with increasing concentration of EDTA from 0.05 to 0.1 mol L^−1^, while it slightly decreased to 93 ± 6 % for 0.2 mol L^−1^ of EDTA. The effect of the eluent flow rate was tested in the range of 0.37–0.70 mL min^−1^. The recovery of Cr(III) (*n* = 3) was 100 ± 0.1 % at a flow rate of 0.37 mL min^−1^, 96 ± 2 % at a flow rate of 0.45 mL min^−1^, 70 ± 2 % at a flow rate of 0.55 mL min^−1^ and 59 ± 2 % at a flow rate of 0.70 mL min^−1^. Then, 0.1 mol L^−1^ EDTA at a flow rate of 0.37 mL min^−1^ was selected as the eluent.

**Fig. 4:**
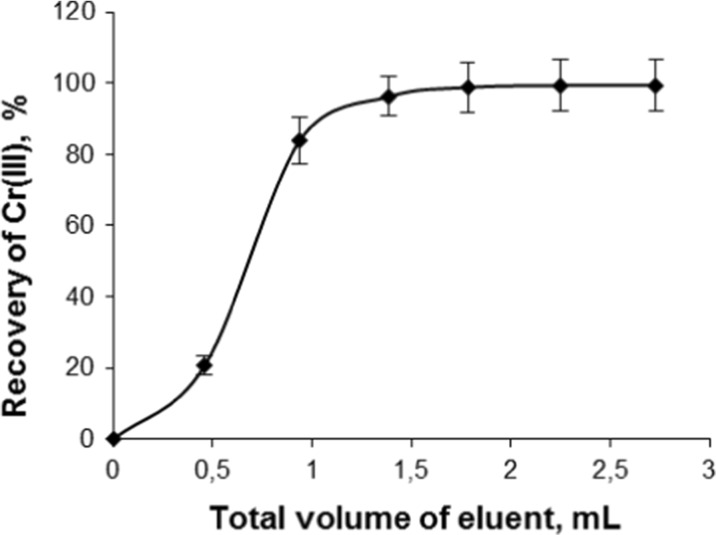
Total recovery of Cr(III) from the polymeric resin functionalized with DPCO ligand with 0.1 mol L^−1^ EDTA (flow rate 0.37 mL min^−1^)

The cumulative recovery of Cr(III) from the column was evaluated using 0.5-mL portions of 0.1 mol L^−1^ EDTA. The fractions of eluate were successively collected, appropriately diluted and analyzed for analyte content by FAAS. It was found that 2 mL of the eluent was sufficient for quantitative (97 %) recovery of analyte from the column (Fig. 4).

The breakthrough capacity of the DPCO-incorporated polymer was determined in flow mode by passing a standard solution of Cr(III) (5 μg mL^−1^) through the columns (0.1 g of polymer). Fractions of effluent (2 mL) were collected and analyzed for chromium concentration by FAAS. The experiment was completed when the efficiency of chromium retention decreased by 20 %. On this basis, the sorption capacity of the polymer for Cr(III) was calculated as 5.4 mg g^−1^.

The results were reproducible for 180 successive sorption-desorption cycles performed on the same portion of polymeric sorbent, which confirmed its suitability for flow procedures. In that respect, the prepared material is superior to other solid sorbents, which retained their adsorption/desorption properties for only five to six analytical cycles (Zhang et al. 2008; Bayramoglu and Arica 2011; Uygun et al. 2013).

### Effect of Other Ions

Selectivity of the polymer functionalized with DPCO towards Cr(III) ions was tested in the presence of other metal ions often present in wastewater (Cu(II), Ni(II), Mn(II), Ca(II)). The distribution ratios (*D*, mL g^−1^) and selectivity coefficients (*α*) of Cr(III) were calculated from the equations described in Leśniewska, et al. (2011) (Table 2). It was found that the selectivity of the sorbent towards Cr(III) ions is much better than towards competitive metal ions.

**Table 2:** Selectivity parameters of the polymeric resin functionalized with the DPCO ligand towards Cr(III) ions in the presence of competitive ions (sample 15 μg of Cr(III) + 15 μg of other metal ion, pH 9, flow rate 0.7 mL min^−1^)

Metal ion, Me	Distribution ratio, *D* (mL g^−1^)		Selectivity coefficient, *α*
	Cr(III)	Me	
Cu(II)	703	140	5.0
Mn(II)	1194	238	5.0
Ni(II)	647	78	8.3
Ca(II)	707	102	7.0

The effect of the presence of Cu(II) ions on the separation of Cr(III) ions on the polymer was also studied. Standard solutions of analyte (10 μg) containing from 10 to 1000 μg of Cu(II) ions were passed through the column. It was found that even 500 μg of Cu(II) ions did not affect neither retention (92–94 %) nor recovery (98–113 %) of Cr(III) from the sorbent. A similar effect was observed in the presence of Mn(II). The recovery of Cr(III) from the sorbent in the presence of 50 μg of Mn(II) (tenfold excess) was in the range 94–109 %. However, during adjustment of sample pH, at solutions containing higher concentrations of Mn(II) ions, the precipitation of manganese hydroxide, accompanied by co-precipitation of Cr(OH)_3_, was observed.

The ability of the polymer to separate chromium species at different oxidation states (Cr(III) and Cr(VI)) was tested for solutions containing both forms of chromium in the ratios Cr(III)/Cr(VI) as 1:1 and 1:5. The solutions were loaded on the column, the next column was rinsed with 2 mL of Milli-Q water, and Cr(III) was eluted with 0.1 mol L^−1^ EDTA and determined by FAAS. The results indicate that the prepared sorbent can be used for the separation of Cr(III) from Cr(VI) species. The content Cr(VI) can be calculated as the difference between total content of chromium and Cr(III) form (Table 3).

**Table 3:** Recovery of Cr(III) from a mixture of Cr(III) and Cr(VI) ions on the polymeric resin functionalized with DPCO ligand (sample: pH 9, flow rate 0.7 mL min^−1^; eluent 2 mL of 0.1 mol L^−1^ EDTA, flow rate 0.37 mL min^−1^; mean value ± SD, *n* = 3)

Model sample	Found mass of Cr(III) ± SD (μg)	Recovery of Cr(III) ± SD (%)	Determined mass of Cr ± SD (μg)	Calculated mass of Cr(VI) (μg)	Recovery of Cr(VI) (%)
9.8 μg of Cr(III) + 9.6 μg of Cr(VI)	8.4 ± 0.2	86 ± 2	19.2 ± 0.4	10.8	113
4.1 μg of Cr(III) + 19.0 μg of Cr(VI)	3.6 ± 0.2	87 ± 2	23.2 ± 0.5	19.6	103

### Analytical Performance

The analytical performance of the method was evaluated under optimized experimental conditions. The efficiency of retention of 5 μg mL^−1^ solution of Cr(III) on the polymer was 95 ± 1 %, while the recovery was 99 ± 5 %. The reproducibility of the developed separation procedure, evaluated for six successive retention and elution cycles and expressed as RSD, was better than 5 %. Under the same conditions, the efficiency of retention Cr(VI) and its elution with Milli-Q water and EDTA solution were tested. It was found that Cr(VI) ions were retained with an efficiency of 32 ± 2 %. Washing up of sorbent with 2 mL of Milli-Q water eluted 59 ± 3 % of Cr(VI). Low recovery of Cr(VI) in EDTA fraction (3 ± 2 %, *n* = 3) indicated efficient removing of interfering ions prior to Cr(III) elution.

The calibration graph for the determination of Cr(III) was constructed by submitting its standard solutions to the separation procedure using 2 mL of the eluent. The graph was linear up to 15 μg mL^−1^ of Cr(III) giving the following regression equation: *y* = 0.0495 × −0.0195 (*r*^2^ = 0.9927). The possibility of separation of Cr(III) from samples containing a high concentration of analyte was confirmed for analysis of 100 μg of Cr(III) (recovery of Cr(III) = 101 ± 5 %). However, in such case, the eluates should be appropriately diluted prior to measurements by FAAS. The limit of detection (LOD) of the method was calculated as the concentration of analyte equals to threefold standard deviation of the absorbance of blank divided by the slope of the calibration graph (3SD_blank_ / *a*), while the limit of quantification (LOQ) was calculated as 10SD_blank_ / *a* (Inczedy et al. 1998). The obtained values were as follows: LOD = 0.030 μg mL^−1^ and LOQ = 0.10 μg mL^−1^.

The accuracy of the method was confirmed by analysis of the reference material of municipal wastewater RES 10.2 with the certified value of the total concentration of chromium (4.55 μg mL^−1^). As was demonstrated earlier (Leśniewska et al. 2015), chromium is present in RES 10.2 only in the Cr(III) form. It was found that the concentration of Cr(III) in the reference material determined after the separation procedure was in good agreement with the certified value for total chromium (Table 4). In terms of sensitivity, the method is comparable to other published methods (Tunceli and Turker 2002; Narin et al. 2006; Rajesh et al. 2008), but it is faster (seven samples/h) and non-laborious.

**Table 4:** Recovery of Cr(III) from real samples on the polymeric resin functionalized with DPCO ligand (sample: pH 9, flow rate 0.7 mL min^−1^; eluent 2 mL of 0.1 mol L^−1^ EDTA, flow rate 0.37 mL min^−1^; mean value ± SD, *n* = 3)

Sample	Found mass of Cr(III) ± SD (μg)	Recovery of Cr(III) ± SD (%)
Sewage^a^ + 14 μg of Cr(III)	15.3 ± 0.5	109 ± 4
Treated sewage^a^ + 14 μg of Cr(III)	13.2 ± 0.4	94 ± 3
Wastewater RES 10.2^b^	4.21 ± 0.16 μg mL^−1c^	93 ± 4

^a^Concentration of Cr below LOD of the method

^b^Wastewater RES 10.2—property value of Cr 4.55 ± 0.055 μg mL^−1^

^c^In concentration units

### Method Application

The method was applied to the determination of Cr(III) in raw and treated municipal sewage. Samples were filtered through a 0.45-μm Supelco membrane filter, adjusted to pH 9 with ammonia solution and left for equilibration. Because the concentration of Cr(III) in analyzed samples was below LOQ of the method, the procedure was applied to samples spiked with 14 μg of Cr(III). The recovery of Cr(III) was in the range of 93–109 % (Table 4), which indicates a significant interference by other metal ions commonly present in such samples. The reproducibility of the separation procedure for different samples was below 3.5 %. This confirmed that the developed SPE method using a resin functionalized with DPCO is suitable for chromium speciation analysis in contaminated samples.

It is worth mentioning that the concentration of chromium in treated sewage samples collected from a municipal sewage treatment plant (Bialystok, Poland) determined by ETAAS was equal to 0.045 μg mL^−1^, which is below the limit established in Poland by the Ordinance of the Minister of Environment (2014).

## Conclusions

A dynamic SPE procedure for the study of speciation of chromium in wastewater and sewage was developed. A new solid material containing the DPCO included in a methacrylate polymeric matrix was designed and prepared for the separation of Cr(III) species. The new sorbent is characterized by high selectivity towards Cr(III) ions, good stability and high sorption capacity (see Table 1). The proposed method was characterized by good reproducibility and low detection limit, comparable to previously published methods (Table 1). Successful validation of the method was performed with the reference material of municipal wastewater RES 10.2. The utility of the method was confirmed in the analysis of complex samples as municipal sewage, while only a few methods presented in the literature have been used for analysis of such samples.

Considering the environmental aspect of our study, it was concluded that the developed method may become a novel analytical tool useful in environmental speciation analysis. The concentration of Cr(VI) can be calculated as the difference between the total concentration of chromium and the Cr(III) form.
